# The neuropsychological phenotype of Chediak-Higashi disease

**DOI:** 10.1186/s13023-019-1049-x

**Published:** 2019-05-06

**Authors:** Talia N. Shirazi, Joseph Snow, Lillian Ham, Greta B. Raglan, Edythe A. Wiggs, Angela C. Summers, Camilo Toro, Wendy J. Introne

**Affiliations:** 1 National Institute of Mental Health, 10 Center Drive, MSC 1274, Bethesda, MD 20892 USA; 2 National Human Genome Research Institute, Bethesda, MD USA

**Keywords:** Chediak-Higashi disease, Intellectual disability, Neuropsychology, Bone marrow transplant

## Abstract

**Background/objectives:**

Chediak-Higashi Disease (CHD) is a rare autosomal disorder, purported to have cognitive and neurological impairments. Prior descriptions of cognitive impairment, however, are solely based on subjective, unstructured observations rather than on formal neuropsychological measures.

**Methods:**

Four pediatric and 14 adult patients with diagnostically confirmed CHD were administered a neuropsychological battery assessing memory, attention, processing speed, psychomotor speed, language fluency, executive function, and general intelligence. Nine of the adult patients received follow-up evaluations to elucidate the longitudinal progression or stability of cognition over time.

**Results:**

Pediatric CHD patients performed within the average range. Adult patients, however, performed below average on nearly all measures administered, and endorsed subjective reports of learning difficulties and poor academic performance in childhood. In particular, patients struggled with memory and psychomotor speed tasks, with 75% or more of patients scoring in the bottom 2.3 percentile in these two domains. No significant declines in cognition were observed among the patients who completed follow-up evaluations (M = 39.90, SD = 8.03 months between visits). Exploratory analyses suggested that adult patients who had classic CHD and previously received bone marrow transplants (BMTs; *n* = 3) exhibited moderately greater cognitive impairment than adult patients who had atypical CHD and had not received BMTs (*n* = 10).

**Conclusions:**

Adult patients with CHD uniformly exhibit deficits in multiple domains, but in psychomotor speed and memory, in particular. Based on their neuropsychological profile, their ability to hold jobs and succeed in school may require support and special accommodations. The source of cognitive deficits is probably multifactorial including central nervous system involvement in CHD, and, for those transplanted, BMT-related side effects and complications. Absence of cognitive decline at three-year follow-up is encouraging but does not exclude progression at a slower time-scale. Future work should elucidate the possible effects and timing of BMT on cognition, as well as the mechanisms driving neuropsychological impairment in CHD.

## Background

Chediak-Higashi Disease (CHD) is an autosomal recessive disorder caused by mutations in the *LYST* gene, with fewer than 500 reported cases worldwide [1]. Central clinical features include immunodeficiency, partial albinism, neutropenia, mild bleeding tendency, and neurodevelopmental disorders during childhood [2]. Without bone marrow transplantation, up to 85% of patients with CHD develop hemophagocytic lymphohistiocytosis (HLH), or the “accelerated” phase of the disease, in childhood, which can result in organ failure and death. Hematopoietic stem cell transplantation has been shown to be an effective treatment correcting the hematologic and immunologic aspects of the disease and reducing the likelihood of the accelerated phase, particularly when conducted prior to the onset of accelerated symptoms [3, 4]. Without bone marrow transplant, fewer than 10% of CHD patients survive past childhood [5]. A subset of patients with a diagnosis of CHD confirmed by molecular genotyping, exhibit attenuated clinical features of CHD (‘atypical’ CHD) and are able to survive into adulthood without hematopoietic cell transplantation and without signs of HLH.

In spite of the progress made towards improving survival and treating the central features of CHD, neurologic deterioration has been noted in adult CHD patients including peripheral neuropathy, motor weakness, ataxia and Parkinsonism [6–12]. Instances of severe neuronal degeneration in the cortex, basal ganglia, and brainstem have also been reported [8, 10, 13]. It is unknown whether this secondary neurological impairment is accompanied by impairments and declines in cognition and everyday functioning.

Though case reports of CHD often include discussion of impairments in cognition and everyday functioning [5, 9, 14–16], the majority do not base such classifications on formal neuropsychological testing. Rather, researchers draw inferences about neuropsychological function on the basis of reported school performance or ability to work. Of the few published case reports of CHD patients where formal neuropsychological tests were administered, all have reported intellectual disability based on IQ [17, 18], but do not provide data on the integrity of individual neuropsychological domains. Observations of intellectual disability have been made in both children and adults with CHD.

Later-life declines in neuropsychological function have been noted in several [5, 10], though not all [6], case studies of CHD patients. The relatively small sample sizes in such studies combined with the lack of longitudinal neuropsychological data make such studies hard to generalize. Additional factors such as consanguineous parentage and whether a patient received a bone marrow transplant may affect cognition [19–22], further contributing to the inability to generalize results from case reports to other patients. The presence of such factors also hinders the ability to isolate the impact of CHD itself on cognition [8, 23].

It is clear that the cognitive presentation of adults with CHD is variable, but no study has systematically assessed the neuropsychological phenotype of CHD patients. Additionally, the lack of longitudinal information about performance on formal cognitive measures means little information is available on neuropsychological progression in CHS patients. Finally, as studies have previously reported global measures of neuropsychological function, the pattern of neuropsychological function across various cognitive domains remains unknown. The purposes of this study are to describe current functioning in a relatively large cohort of CHD patients as measured by traditional neuropsychological measures, to examine the progression of symptoms over time in those cases in which follow-up data allows, to determine the effect of age on functioning in CHD patients cross-sectionally by comparing adult and pediatric patients, and to evaluate the relationship between cognitive functioning and clinical markers of syndrome severity.

## Method

### Recruitment

Patients were recruited between 2005 and 2016 to participate in a National Human Genome Research Institute Institutional Review Board-approved study on Chediak-Higashi Disease (NCT identifier NCT00005917). Patients were also referred to the study by the CHD patient support group, the internet (Clinicaltrials.gov), and through national meetings. All patients enrolled in the study were confirmed to have CHD by observing giant inclusions within leucocytes and molecular and cell biologic studies. Informed consent was obtained from all patients. See Table 1 for demographic information.

### Neuropsychological procedures

Formal neuropsychological assessments were conducted at the National Institutes of Health (NIH) by licensed clinical neuropsychologists (JS and EAW), psychologists, or by trained psychometrists. Pediatric patients were administered a measure of general intelligence and parents filled out a self-report form on behavioral and emotional functioning (see Table 2). Adult patients were administered a comprehensive neuropsychological battery assessing memory, attention, processing speed, psychomotor speed, language fluency, executive function, and general intelligence (see Table 3). When time permitted, patients and informants also filled out validated self-report measures of executive function. In addition, some patients completed self-report measures of depression and anxiety in order to assess mood. In the event that these measures were not completed at the initial assessment (i.e., informant did not return the form or the form was not administered), we obtained this information from a subsequent visit. We provide results for these behavior and mood measures gathered at baseline or at subsequent visits in Table 4. As not all tests were administered for all participants, we report the number of patients who completed each test along with its descriptive statistics. Descriptive statistics are reported as T-scores, which have a mean of 50 and standard deviation (SD) of 10. Variables based on tests conducted at the NIH that are typically reported using other statistics (e.g., IQ scores typically are reported as standard scores have a mean of 100 and SD of 15) were linearly transformed to T-scores based on the normal distribution. Lower T-scores indicate poorer performance with the exception of FrSBe and CBCL scores (informant reports for adults and children, respectively) where higher scores indicate greater dysfunction. *T*-scores on the Conners’ Continuous Performance Test-II were reverse-scored so that lower *T*-score reflects greater impairment. As CHD is a developmental disorder, we chose to minimally demographically correct the T-scores. All T-scores were demographically corrected for age, while a select few were corrected for additional demographic variables as required by various scoring software (see Table 3 for further information). Normative values were obtained through widely used published and commercially available norms derived from generally large and representative samples in the United States. Means and SDs for individual measures were calculated using data from patients’ initial neuropsychological evaluations. The subset of patients who completed reevaluations did so at approximately one-year intervals. Pediatric patients were administered a measure of general intelligence and parents filled out a self-report form on behavioral and emotional functioning (see Appendix for list of all measures).

**Table 1:** Adult CHD patient demographics

Patient ID	Age at initial visit	LYST variants	CHD diagnosis	Transplant (age at transplant)	Psychotropic Medications
CHD-19*	17	p.R1104X p.G3408R	Atypical	No	None
CHD-27	21	Unknown Unknown	Classic	Yes (3 years)	None
CHD-26	21	p.R503X p.G3309S	Atypical	No	None
CHD-18*	22	p.R1104X p.G3408R	Atypical	No	None
CHD-35	22	p.E489DfsX78 p.E489DfsX78	Classic	Yes (6 months)	None
CHD-24†	23	p.A1454D p.Y1687X	Atypical	No	None
CHD-23†	26	p.A1454D p.Y1687X	Atypical	No	None
CHD-17	28	p.L1425YfsX1 p.E2810K	Atypical	No	None
CHD-5	28	p.V2651F Unknown	Atypical	No	Lithium, Lamictal, Zoloft (off Lithium by second visit)
CHD-30	29	Unknown Unknown	Classic	Yes (10 years)	None
CHD-20	31	p.R1104X p.R1104X	Classic	No	None
CHD-31^#^	33	p.N3276_T3277del p.N3276_T3277del	Atypical	No	None
CHD-33^#^	38	p.N3276_T3277del p.N3276_T3277del	Atypical	No	None
CHD-32^#^	43	p.N3276_T3277del p.N3276_T3277del	Atypical	No	None
Patient ID	Available Prior Evaluation Results	Prior Neuropsychological Diagnoses	Assistance in school	Initial Visit Occupation	Current Occupation
CHD-19*	Est. FSIQ = 78 (at 9 yrs. 7 mos)	Diagnosed ADHD	IEP grades 2–7; special education courses	Student	Unemployed
CHD-27	None	Diagnosed learning disability	IEP grades K-12; special education courses; repeated kindergarten	Community College student	Day care provider
CHD-26	None	Diagnosed learning disability	IEP; special education courses	Unemployed	Unemployed
CHD-18*	Est. FSIQ = 101 (at 6 yrs. 6 mos)	Diagnosed ADHD	Unknown	Department store warehouse worker	Unemployed
CHD-35	None	Diagnosed learning disability	IEP grades 2–12	Odd jobs for family members	Odd jobs for family members
CHD-24†	None	Diagnosed reading disorder and ADD	IEP grades 3–12	Cleaning and maintenance work	Unemployed
CHD-23†	None	None	None reported	Food service and janitorial position	Unemployed
CHD-17	None	None	Special education courses	Unemployed	Unemployed
CHD-5	None	None	IEP grades 9–12	Nursing assistant	Unemployed
CHD-30	Est. FSIQ = 75 (at 18 yrs)	Diagnosed learning disability; borderline ADHD	No special accommodations; private tutoring	Cashier at family business	Unemployed
CHD-20	None	Diagnosed learning disability	Unknown	Unemployed	Deceased
CHD-31^#^	None	None reported	Unknown	IT	Unemployed
CHD-33^#^	None	Diagnosed learning disability and ADHD	None reported	Office support staff	Unemployed
CHD-32^#^	None	Diagnosed learning disability; ADHD	Special tutoring in school prior to age 7	Unemployed	Unemployed

Superscripts indicate sibling sets

**Table 2:** Table with results for pediatric subsample

	*n*	Mean (SD)
General Intelligence		
WPPSI-III Verbal IQ	3	47.8 (9.6)
WPPSI-III Performance IQ	4	46.3 (6.8)
WPPSI-III FSIQ	3	45.8 (6.8)
WRAT-4 Word Reading	2	44.0 (10.4)
WRAT-4 Spelling	2	41.0 (1.4)
WRAT-4 Math Computation	2	43.0 (0.5)
Behavioral and Emotional Functioning^a^		
CBCL Emotionally Reactive	3	50.7 (0.6)
CBCL Anxious/Depressed	3	50.3 (0.6)
CBCL Somatic Complaints	3	55.0 (8.7)
CBCL Withdrawn	3	56.3 (9.2)
CBCL Sleep Problems	2	52.0 (4.5)
CBCL Attention Problems	3	57.3 (4.5)
CBCL Aggressive Behavior	3	52.0 (2.7)
CBCL Internalizing Problems	3	50.0 (5.2)
CBCL Externalizing Problems	3	52.0 (5.0)
CBCL Total Problems	3	49.0 (5.0)
CBCL Stress Problems	3	53.0 (2.0)
CBCL Affective Problems	3	51.3 (1.2)
CBCL Anxiety Problems	3	50.3 (0.6)
CBCL Pervasive Developmental Problems	3	55.0 (4.6)
CBCL ADHD Problems	3	54.3 (4.9)
CBCL Oppositional Defiant Problems	3	51.3 (1.2)

^a^Higher scores indicate greater dysfunction

### Additional measures

Information on the majority of patients’ premorbid functioning was obtained through interviews with the patient and informants. For the three adult patients who reported undergoing neuropsychological testing prior to enrolling in the current protocol, results from previous testing sessions were obtained. Patients underwent comprehensive neurologic examinations as well, though discussion of such measures is beyond the scope of the present manuscript and has been previously reported on a subset of the present cohort [12]. However, here we do present data on MRI-based cerebellar and cerebral atrophy for the full adult cohort that received scans around the time of neuropsychological evaluation (see Table 5).

**Table 3:** T-scores (M = 50, SD = 10) on formal neuropsychological tests

	*n*	Mean (SD)	Percent scoring in bottom 16th percentile of population	Percent scoring in bottom 2.3 percentile of population
General Intelligence				
Wechsler FSIQ^a^	12	32.9 (8.1)	67%	42%
WRAT-4 Word Reading^a^	12	40.1 (8.7)	42%	17%
WRAT-4 Spelling^a^	8	44.3 (9.5)	38%	13%
WRAT-4 Math Computation^a^	9	33.2 (6.0)	100%	22%
Executive Function				
WCST Total Errors^a^	9	30.6 (8.6)	100%	44%
WCST Perseverative Responses^a^	9	34.6 (8.8)	56%	22%
Attention/Working Memory				
WAIS-III Digit Span^a^	13	45.9 (7.7)	15%	0%
WAIS-III Letter-Number Sequencing^a^	10	40.1 (7.0)	40%	0%
CPT-II Omissions^b^	9	19.0 (19.5)	78%	78%
CPT-II Commissions^b^	9	42.7 (8.4)	22%	11%
CPT-II Reaction Time^b^	9	38.3 (9.5)	67%	11%
CPT-II Reaction Time Standard Error^b^	9	29.2 (15.6)	78%	56%
Processing Speed				
WAIS-III Digit-Symbol Coding^a^	13	31.5 (6.2)	86%	38%
WAIS-III Symbol Search^a^	13	36.9 (5.2)	54%	0%
Language Fluency				
COWA FAS^c^	8	36.8 (7.0)	67%	13%
COWA Animals^c^	8	35.0 (8.3)	50%	38%
Learning and Memory				
NAB Memory Index^a^	12	26.4 (12.3)	83%	75%
BVMT-R Learning^a^	5	23.2 (7.7)	100%	80%
BVMT-R Memory^a^	5	25.2 (10.1)	80%	80%
HVLT-R Learning^a^	7	25.1 (8.1)	86%	86%
HVLT-R Memory^a^	7	28.4 (9.0)	71%	57%
Psychomotor Speed				
Grooved Pegboard (dominant)^c^	8	16.4 (9.3)	100%	100%
Grooved Pegboard (nondominant)^c^	8	15.4 (9.8)	100%	100%

^a^ T-score demographically corrected for age only

^b^ T-score demographically corrected for age and gender

^c^ T-score demographically corrected for age, education, race, and gender

FSIQ scores were linearly transformed from standard scores to *T*-scores based on the normal distribution. *T*-scores on the CPT-II were scored so that lower T-score reflect greater inattention (i.e., scoring program-derived output was transformed via the formula 50 - (score-50))

## Results

### Patients

Four pediatric patients (1 male, 3 female; aged 4–5) completed a neuropsychological evaluation. All pediatric patients were diagnosed with CHD during infancy, and all had received at least one BMT prior to testing.

Fourteen adult patients (10 male, 4 female; aged 17–43) also completed an initial neuropsychological evaluation, and of those, nine patients (64.29%) had at least one subsequent reevaluation. Adult patients were on average 27.3 years old (SD = 7.2) at initial evaluation. Age at CHD diagnosis ranged from birth (*n* = 7, 50% of total sample) to 43 years old. Three patients had previously received a BMT (at 6 months, 3 years, and 10 years of age) and were diagnosed with classic CHD. One patient was diagnosed with classic CHD but never received a BMT. The remaining patients had not received a BMT previously and were diagnosed with atypical CHD. Many patients had eye or vision conditions such as nystagmus, color vision deficits, myopia, hyperopia, and strabismus. For most of these patients, conditions were mild and treated or corrected and it is not believed that vision problems affected their test results. A few patients had significant vision problems that progressed over subsequent visits. For those patients who had vision difficulty that could have potentially affected test performance, select tests with a vision component were omitted from the battery.

Many patients also had motor difficulties, such as tremors and mild upper extremity weakness. However, these difficulties would not affect the majority of the administered cognitive tests, except those of psychomotor speed and information processing. Because tests of psychomotor speed and information processing aim to measure brain-related motor abilities, it would be inappropriate to exclude scores from patients with motor impairments, as these scores define the neurocognitive phenotype of CHD. Medications taken at the time of the initial assessment are listed by patient in Table 1. There was only one adult patient who was on Lithium and Zoloft for Bipolar Disorder. No other patients had a psychiatric diagnosis (other than a history of a learning disorder or ADHD) and no one was excluded on the basis of comorbid conditions or medication use. Three sets of siblings participated in the study (two sibling pairs and one sibling trio), while the remaining 7 patients were from independent families. Additional demographic information can be found in Table 1.

**Table 4:** T-scores (M = 50, SD = 10) on performance and rating scales

	Baseline *n*	Baseline Mean (SD)	Baseline + Follow-up *n*	Baseline + Follow-up Mean (SD)
Behavioral and Emotional Functioning^a^				
FrSBe Family Before Apathy	9	63.6 (21.4)	12	68.0 (20.1)
FrSBe Family Before Disinhibition	9	46.2 (11.0)	12	50.2 (17.4)
FrSBe Family Before Executive Dysfunction	9	59.9 (17.1)	12	64.0 (16.5)
FrSBe Family Before Total	9	50.2 (22.4)	12	56.8 (23.1)
FrSBe Family After Apathy	9	73.4 (29.3)	12	75.6 (25.4)
FrSBe Family After Disinhibition	9	49.2 (14.3)	12	52.6 (19.0)
FrSBe Family After Executive Dysfunction	9	62.1 (19.8)	12	66.4 (19.2)
FrSBe Family After Total	9	62.3 (21.2)	12	66.3 (20.6)
FrSBe Self Before Apathy	7	54.3 (11.2)	10	54.0 (9.5)
FrSBe Self Before Disinhibition	7	43.1 (12.3)	10	46.0 (11.5)
FrSBe Self Before Executive Dysfunction	7	51.3 (14.8)	10	52.6 (14.8)
FrSBe Self Before Total	7	49.7 (15.8)	10	51.4 (13.3)
FrSBe Self After Apathy	7	57.1 (18.5)	11	54.7 (16.4)
FrSBe Self After Disinhibition	7	41.7 (10.9)	11	45.2 (10.6)
FrSBe Self After Executive Dysfunction	7	50.9 (16.5)	11	49.7 (16.9)
FrSBe Self After Total	7	49.9 (18.3)	11	49.5 (17.3)
Mood^a,b^				
Beck Depression Inventory – II	10	6.4 (6.1)	12	6.6 (6.4)
Beck Anxiety Inventory	6	3.2 (2.6)	11	4.8 (3.7)

^a^ Higher scores indicate greater dysfunction

^b^ Raw total score out of 63

Baseline = data gathered from first visits only; Baseline + Follow-up = data gathered from first or subsequent visits

### Neuropsychological findings from the pediatric subsample

Performance on the Wechsler Preschool and Primary Scale of Intelligence-III suggested low, but average abilities across individual subtests as well as on the composite measures of Verbal IQ (M = 47.78, SD = 9.65), Performance IQ (M = 46.8, SD = 6.76), and FSIQ (M = 45.78, SD = 6.84). Scores on the Wide Range Achievement Test-4, a measure of academic function, were low but within normal limits as well. At time of initial testing, no parents indicated any concerns about possible ADHD; however, scores on two scales of attention on a parent-report measure of behavioral and emotional functioning (Child Behavior Checklist) indicated slightly elevated (though within normal limits) levels of attentional difficulties. Other subscales were generally within normal limits (see Table 2).

**Table 5:** Adult patient clinical neuroimaging findings and average T-scores

	Neuroimaging		
Patient ID	Cerebellar Atrophy^a^	Cerebral Atrophy	Average neuropsychological T-score (M = 50, SD = 10)
CHD-17	0	0	43
CHD-27	+	0	31
CHD-26	+	0	27
CHD-19	+	0	40
CHD-24	0	+	32
CHD-5	Not scanned	Not scanned	38
CHD-23	+	++	35
CHD-20	++	+++	30
CHD-30	+++	+++	30
CHD-33	0	+	32
CHD-31	0	+	43
CHD-18	+	0	42
CHD-32	++	+++	30
CHD-35	0^	0^	30

^a^Neuroimaging revealed that all individuals had cerebellar hypoplasia

0 = no abnormality, + = mild impairment, ++ = moderate impairment, +++ = severe impairment, ^=based on non-NIH scan

### Neuropsychological findings from the adult subsample

#### Early academic functioning

Eight adult patients (57.14%) reported having an individualized education program (IEP) or specialized academic accommodations during school, three (21.43%) did not have an IEP or specialized academic accommodations, and this data was unavailable for the remaining three patients. Eight patients (57.14%) went on to pursue college education, although only one of these eight completed a Bachelor’s degree. Six patients (42.86%) were previously diagnosed with ADHD, of which none were on medication at time of initial testing, though the reasons for not being on medication are mostly unknown. Two patients took medication for ADHD previously, but discontinued due to side effects (e.g. headaches, vomiting). Seven patients (50%) were previously diagnosed with a learning disorder. Two of the three patients who had intelligence testing prior to enrolling in the current protocol had estimated full-scale IQs (FSIQ; M = 100, SD = 15) in the ‘borderline’ range (i.e. between 70 and 79), while one had an estimated FSIQ of 101. All 14 adult patients completed high school, and all but one received a standard high school diploma (as opposed to a special education diploma).

#### Intelligence and academic achievement

Based on the assessment at NIH, a FSIQ was calculated for 12 adult patients. Four patients (28.57%) had an FSIQ between the mean and one SD below, one (7.13%) between one and two SD below the population-based mean (bottom 16th percentile), and seven (50%) greater than 2 SD below the mean (bottom 2.3 percentile; overall M = 32.8, SD = 8.0). Performance on Wide Range Achievement Test-4 subtests was variable across subjects and across subtests. All adult participants that were administered Math Computation (*n* = 9) scored in the bottom 16th percentile (overall M = 33.2, SD = 6.0). Patients appeared to perform better on the Word Reading subtest (*n* = 12), with only five (35.71%) receiving a score in the bottom 16th percentile (overall M = 40.1, SD = 8.7). Similarly, of the eight patients who underwent the Spelling subtest, only three received a score in the bottom 16th percentile (overall M = 44.3, SD = 9.35).

#### Formal neuropsychological tests of specific domains

Table 3 displays the number of participants, mean T-scores, standard deviations, and percent of participants scoring in the bottom 16th and bottom 2.3 percentile of the population for neuropsychological tests that were administered to at least five participants. Overall, on average participants performed below average on all measures with the exception of Wechsler Adult Intelligence Scale-III Digit Span. The greatest impairments were observed on the Grooved Pegboard, a test of psychomotor speed, where all participants scored in the bottom 2.3 percentile. Participants also performed significantly below average on measures of learning and memory and attention. Overall NP (neuropsychological) performance was not significantly associated with participant age (β = − 0.1, *t*(13) = − 0.30, *p* = 0.75), suggesting that differences in NP performance observed across participants was not influenced by differences in participant age.

**Fig. 1:**
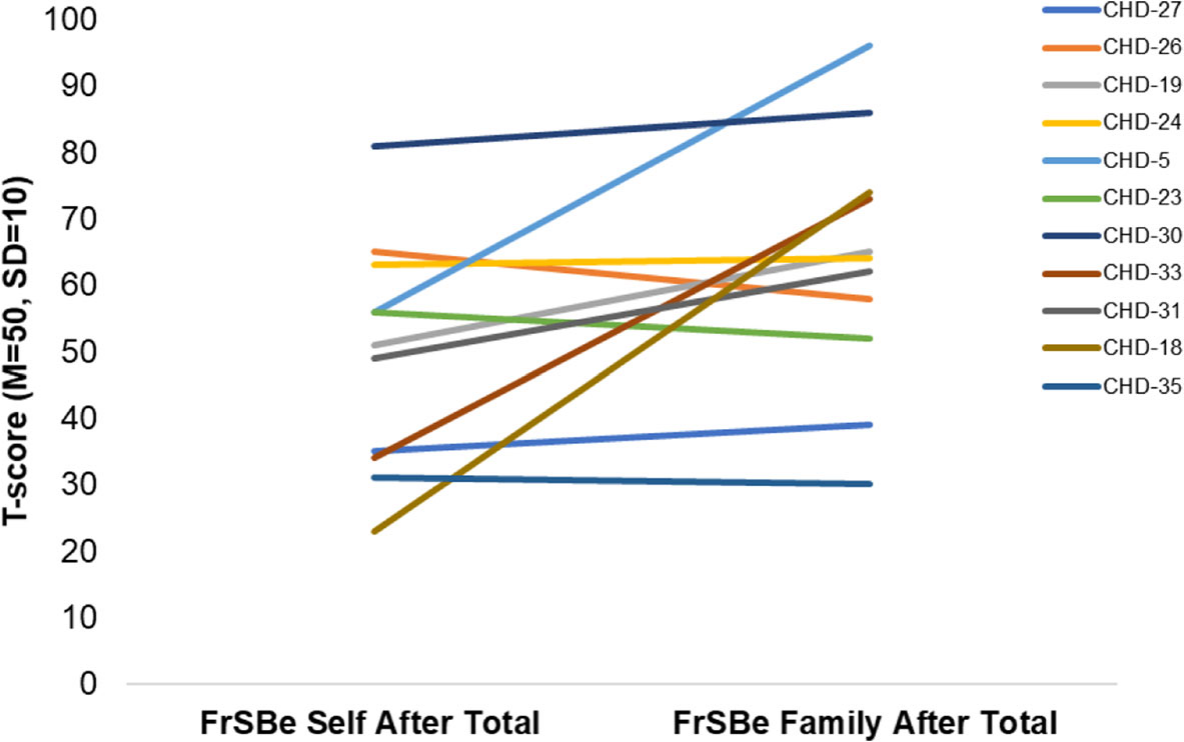
Differences in T-scores between self-reported and informant-reported behaviors related to executive function. Higher scores indicate greater dysfunction. Three patients were excluded due to incomplete data. There was a significant difference between self-reported behaviors (*M* = 49.45; *SD* = 17.31) and informant-reported (*M* = 63.55, *SD* = 19.11); *t*(10) = 2.33, *p* = .042, *d* = .70

In addition to being examined using formal neuropsychological tests, executive function was further examined using Frontal Systems Behavior Scale (FrSBE) informant (*n* = 9) and self-reports (*n* = 7; see Table 4). Scores on the informant-report apathy (M = 73.4, SD = 29.3) and executive dysfunction (M = 62.1, SD = 19.8) subscales were elevated (with elevated scores indicative of impairment) for six and four patients, respectively; elevated scores on the disinhibition subscale were only observed in two patients (M = 49.2, SD = 14.3). Incorporating those who had informant (*n* = 12) or self-report (*n* = 11) data collected at a later evaluation, scores on informant-report apathy (M = 68.0, SD = 20.1) and executive dysfunction (M = 64.0, SD = 16.5) subscales were elevated for nine and seven patients, respectively; elevated scores on the disinhibition subscale were observed in four patients (M = 52.6, SD = 19.0). The mean overall informant FrSBe score was slightly elevated at 62.3 (SD = 21.2), and more elevated with inclusive data (M = 66.3, SD = 20.6). Comparing informant (M = 63.55, SD = 19.11) versus self-report scores (M = 49.45; SD = 17.31) on the subscales, informants reported significantly greater impairment than patients; t(10) = 2.33, *p* = .042, d = .70 (see Fig. 1). Data collected at baseline and at follow-up evaluations was used for this comparison in order to maximize sample size; however, this finding should be interpreted with caution due to the very small sample (*n* = 11).

#### Mood

Mood was evaluated using the self-report, 21-item Beck Depression Inventory-II (BDI-II; *n* = 10) and Beck Anxiety Inventory (BAI; *n* = 6; see Table 4). Depression (M = 6.4, SD = 6.1) and anxiety scores (M = 3.2; SD = 2.6) were both in the minimal range (0–13; 0–7) at the first evaluation, and when incorporating those who had data collected at a later evaluation (BDI-II: *n* = 12; M = 6.6, SD = 6.4; BAI: *n* = 11; M = 4.8; SD = 3.7). Across follow-up evaluations, depression symptoms remained in the minimal range (0–13), and anxiety symptoms were in the minimal (0–7) to mild range (8–15).

#### Longitudinal cognitive functioning

**Fig. 2:**
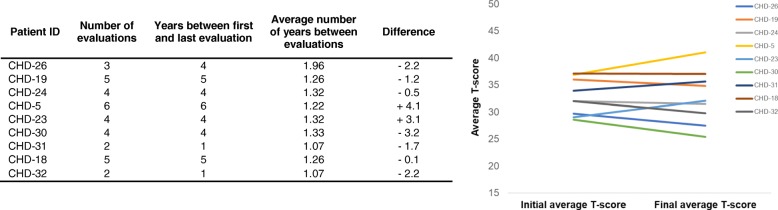
Changes in average T-scores between initial and final visits for adult patients who completed more than one evaluation. Note that all T-scores are not based on the same battery of test for all patients

Of the 14 patients, nine had repeated evaluations for the purposes of this study with the average number of follow-up evaluations being 2.86 (SD = 1.79, range = 2–6), with evaluations conducted approximately 1 year apart. Data from patients’ first administration of a particular test was compared to that from their last administration to elucidate whether patients evidenced a decline in functioning over the time of their participation in the study. We report results on the tests for which at least six patients had longitudinal data. Of the 25 variables examined, three exhibited changes over time. Wechsler Adult Intelligence Scale-III arithmetic scores exhibited improvements between first and last administration (*t*(5) = 3.80, *p* = 0.01), while Conners’ Continuous Performance Test-II omissions and commissions exhibited declines (*t*(7) = 2.63, *p* = 0.03 and *t*(7) = 3.78, *p* < 0.01, respectively). Patients’ average NP T-scores did not decline across evaluations (*t*(8) = 0.07, *p* = 0.94; see Fig. 2).

#### Transplant, diagnosis and cognitive functioning

Exploratory analyses were conducted to detect potential differences between adult patients with a diagnosis of classic CHD and previous BMT (*n* = 3) and with a diagnosis of atypical CHD and no previous BMT (*n* = 10). As there was only one adult patient with a diagnosis of classic CHD that did not receive a BMT, this patient was excluded from analyses. Average NP T-scores in patients with classic CHD and previous BMT (M = 30.27, SD = 0.90) were significantly lower than those of patients with atypical CHD and no previous BMT (M = 35.77, SD = 5.95; *t*(11) = 2.95, *p* = 0.01). Additionally, T-scores on the Wide Range Achievement Test-4 Spelling subtest were 14 points higher in patients without BMTs (*t*(7) = 3.04, *p* = 0.03), T-scores on the Wide Range Achievement Test-4 Spelling Math Computation subtest were 10.5 points higher (*t*(8) = 3.81, *p* = 0.04), and T-scores on the Wechsler Adult Intelligence Scale-III Block Design subtest were 11 points higher (*t*(7) = 2.46, *p* = 0.04). No other significant differences were detected between the two groups on any individual measure.

## Discussion

Increased survival rates due to early BMTs, as well as the identification of individuals with milder forms of the disorder, have afforded the ability to study the manifestations of CHD in adulthood, and their longitudinal progression. The present study represents the first to use formal neuropsychological tests to evaluate the cognitive phenotype of CHD. Results from pediatric patients (all of whom previously received BMTs) suggest a lack of impairments in cognition and emotional functioning. Results from adult patients (the majority of whom did not previously receive BMTs), on the other hand, suggest impairments across almost all cognitive domains measured, longitudinal stability of such impairments across the study period, and a potential influence of diagnosis (i.e., classic versus atypical CHD) or prior BMT on cognition.

Though results from formal neuropsychological testing during childhood were not available for the majority of adult patients, reports of adult patients’ educational history are generally suggestive of early neuropsychological impairments. More than 75% of patients were diagnosed with a learning disability, ADHD, or both as a child; in contrast, about 8 % of children in the US population are diagnosed with a learning disability, and about 5 % are diagnosed with ADHD [27]. Interestingly, data from the pediatric sample in the present study does not support the notion of increased neuropsychological impairment in pediatric CHD patients, though it may be that such impairments are not readily apparent until later on in formal schooling. On the other hand, early identification, intervention, and BMT may protect against the presumed deleterious effects of CHD and its concomitant immunological complications on cognition. That all pediatric patients received at least one BMT in infancy as compared to just three of adult patients may contribute to the discrepancies observed in childhood neuropsychological function. Interestingly, the seemingly protective effect of BMT in pediatric patients was not observed in adult patients, as patients with classic CHD and prior BMTs exhibited significant neuropsychological impairments. It is possible that when adult patients received BMTs, there was significantly greater neurotoxicity in the chemotherapy preparative procedures, whereas this neurotoxicity has been reduced and is not experienced in patients receiving BMTs more recently.

The greatest and most consistent impairments observed in adult patients were in psychomotor speed, where mean scores for both dominant and nondominant hands fell in the bottom 0.1 percentile. The frank psychomotor impairments described here are concordant with previously reported motor impairments in CHD such as Parkinsonism [6, 10–12] and dysmetria [12], as well as with cerebellar atrophy, cerebellar hypoplasia, and cerebral atrophy noted in subsets of CHD patients [12; see also Table 5]. Abnormalities have been more consistently reported in the posterior fossa of CHD patients [12], which may also modulate psychomotor function. Neuropsychological tests of other cognitive functions may also rely on psychomotor functioning to a limited degree and could therefore also be somewhat affected. Although our limited sample size makes it difficult for us to draw any firm quantitative conclusions regarding cognition-to-neuroimaging correlations, the data presented in Table 5 suggests that individuals with the greatest degree of cerebellar and cerebral atrophy have a trend toward greater neurocognitive impairment based on their average T-score.

In the present study, two measures of information processing speed (Wechsler Adult Intelligence Scale-III Digit Symbol Coding and Symbol Search) as well as two measures of attention (Conners’ Continuous Performance Test-II RT and RT Standard Error) requiring motor output were administered. Scores on these tests may be depressed due to psychomotor impairments, potentially obscuring true functional abilities in attention and information processing speed. However, scores relatively independent of psychomotor demands were also below average. Second to that of psychomotor function, the greatest difficulties were observed in tests of learning and memory, with both visual memory (assessed using the Neuropsychological Assessment Battery and Brief Visuospatial Memory Test-Revised) and verbal memory (assessed using the Neuropsychological Assessment Battery and Hopkins Verbal Learning Test-Revised) exhibiting similar levels of impairment, with mean scores in the bottom 2.3 percentile. Future work should examine neuropsychological and neuroimaging data in concert to elucidate the anatomical basis of the cognitive profile of CHD observed in the present study.

Progressive cognitive decline was not observed in the present study. While it may be that the trajectory of cognitive decline in CHD patients is similar to that of controls, it is also possible that these trajectories differ, but that the present study was not able to detect these differences.

It is possible that CHD patients do in fact experience cognitive decline, but that it occurs over a course of a time span greater than that of the present study; for example, patients may experience a period of unchanging cognitive function, followed by a rapid decline. It also may be that CHD patients experience an ‘accelerated aging’ process such as that proposed in HIV patients [24], where cognitive decline is experienced more rapidly and at an earlier age as compared to healthy controls. As all but one patient in the present sample were younger than 40, we were unable to examine cognition at ages where decline would be expected. The mild level of neuropsychological impairment detected among pediatric CHD patients relative to that observed in adult patients may mirror general neurologic involvement in CHD, wherein subtle abnormalities are noted in childhood, but are followed by a period of progressive degeneration in early adulthood, wherein abnormalities and impairments become increasingly pronounced. Alternatively, it is possible that CHD involves an early curtailing of mental development or plateauing, as opposed to loss of functioning.

Although other chronic multisystemic diseases may also involve progressive degeneration in neurologic function and in some cases in cognitive function, the proposed two-stage model here may be unique to CHD. Our data suggest subtle and mild deficits in childhood, followed by progressive neurological and cognitive decline in adulthood, which is a pattern atypically observed in other chronic multisystemic diseases. It is important to note, however, that in absence of longitudinal data following CHD patients from childhood into adulthood, we cannot be certain about the exact pattern of neuropsychological decline in this clinical population. It should also be pointed out that the extent and pattern of deficits observed in adult CHD patients --that of disproportionate and severe psychomotor and memory difficulties -- while not completely unique to CHD, as it is found in other conditions such as Parkinson’s Disease and subcortical dementias, is not found in many other multisystemic diseases.

Our finding of low neuropsychological function in all adult patients serves as a cautionary note for doctors, family members, and others involved in the care of CHD patients. However, the present study may be viewed somewhat positively insofar as there was no significant developmental deficits in our transplanted pediatric patients nor evidence of rapid decline in our adult sample. Patients with CHD in high school may experience greater difficulty in school than their unaffected counterparts, but it is possible for patients to complete high school when provided with special accommodations, and even when not provided with such accommodations. Greater difficulties may be experienced during the pursuit of postsecondary education, where academic support may not be as readily available. Several patients were able to find employment despite neuropsychological impairments and a lack of postsecondary education, but the majority of patients who were employed at their first evaluation were no longer employed at their last. Though this would potentially suggest a worsening of cognition over time, no longitudinal decreases in cognition were observed. It is possible that the trend of previously employed patients being unemployed at follow up can be explained by a progression of the physical features of CHD, rather than by cognitive features, though the extent to which physical and cognitive symptoms uniquely contribute to impairments in functioning are difficult to estimate. It is also positive, and perhaps unexpected, that despite their impairments in cognition and in everyday functioning, CHD patients report minimal amounts of depression and anxiety. Though FrSBe subscale scores indicate an elevated level of self-reported apathy, which is typically a core feature of depression, this heightened apathy does not manifest in elevated depression scores. As indicated by low executive functioning across traditional neuropsychological tests and the FrSBe, as well as by discrepant FrSBe scores in patients and informants, self-awareness among CHD patients may be low, and it may be this low self-awareness that in part explains low levels of reported depression and anxiety.

The present study was not without limitations. First, that the majority (11/14) of adult patients in the present study did not have BMTs suggests most of our patients may have had a milder or simply unique variant of CHD, and that their results may therefore not generalize well to the broader population of CHD patients who typically require BMTs for survival. Second, the fact that not all patients received the same testing battery contributes to the low number of patients having completed any one particular test. Third, because the adult patients with classic CHD received BMTs and no patients with atypical CHD received BMTs, we are unable to disentangle the unique effects of BMT and CHD diagnosis (i.e. classic versus atypical) on cognition. Despite these limitations, the present study represents the first attempt at formally testing multiple cognitive domains in the CHD population, whereas prior research on neuropsychological function in CHD has been largely derived from case reports and informal measures of cognition.

Our results suggest several avenues for future work. First, longitudinal studies beginning in childhood and continuing into adulthood should be employed to better elucidate the trajectory of neuropsychological function in CHD patients. Such studies involving deep phenotyping would afford the ability to examine possible mechanisms contributing to cognitive impairment in CHD as the mechanisms by which mutations in the *LYST* gene in CHD deleteriously affect cognition and CNS function more generally have not yet been identified. Longitudinal studies would also allow physicians and caregivers to investigate the best academic resources and/or medications to provide to a patient group with a high prevalence of ADHD and learning disabilities. Finally, prospective pre- and post-BMT studies should be conducted to evaluate the effect of BMT on cognition in CHD patients, as some prior work has suggested a deleterious effect of BMT [25, 26]. As the majority of CHD patients require BMT for survival past childhood and adolescence, the ability to accurately identify the risks of BMT may significantly impact the post-BMT care and services patients receive.

## Conclusion

Adults with CHD exhibit cognitive impairment across a wide range of neuropsychological domains, and these impairments may be compounded in patients with classic CHD who previously received BMTs. Longitudinal analyses suggest that there is little evidence of cognitive decline in adult CHD patients over a period of several years. Pediatric CHD patients with prior BMTs performed within the average range, but long-term follow-up analyses are required to elucidate the trajectory of cognition in CHD.

## Appendix

**Table 6:** Assessment battery

Domain	Assessment
FSIQ	WASI
	WAIS-III
	WPPSI-III (pediatric sample only)
Information Processing	Digit-Symbol Coding (WAIS-III)
	Symbol Search (WAIS-III)
Attention	Digit Span (WAIS-III)
	Conners’ Continuous Performance Task-II (CPT-II)
	-Omissions
	-Commissions
	-Reaction time
	-Reaction time standard error
Language Fluency	Controlled Oral Word Association Task (COWA) - FAS
	Controlled Oral Work Association Task (COWA) - Animals
	Boston Naming Test
Learning and Memory	Neuropsychological Assessment Battery (NAB) Memory Module
	Hopkins Verbal Learning Test (HVLT-R)
	-Total recall
	-Delayed
	Brief Visuospatial Memory Test (BVMT-R)
	-Total recall
	-Delayed
Psychomotor Speed	Grooved Pegboard (Dominant Hand)
	Grooved Pegboard (Nondominant Hand)
Executive Functioning	Wisconsin Card Sorting Task (WCST)
	-Errors
	-Perseverative responses
	Frontal Systems Behavior Scale (FrSBe)
	-Total (informant report)
	-Total (self-report)
Emotional and Behavioral Functioning	Child Behavior Checklist (CBCL; pediatric sample only)

Not all patients were administered all the above tests. However, on their first visits, all patients were administered at least one assessment of information processing, attention, learning and memory, and subtest from the WASI/WAIS. Additionally on their first visits, all patients but one were administered at least one test of executive functioning, psychomotor speed, and language fluency. Patients with follow-up evaluations were administered similar batteries across evaluations

## Abbreviations

BMT: Bone marrow transplant
CHD: Chediak-Higashi Disease
FSIQ: full-scale IQ
HLH: hemophagocytic lymphohistiocytosis
IEP: individualized education program
NIH: National Institutes of Health
NP: neuropsychological
SD: standard deviation
